# The effects of environmental stress on third molar agenesis in humans and its population differences

**DOI:** 10.1537/ase.251105

**Published:** 2026-01-27

**Authors:** Retsu Katsuyama

**Affiliations:** 1 Laboratory of Physical Anthropology, Graduate School of Science, Kyoto University, Kyoto, Kitashirakawa-oiwakecho, Sakyo-ku, Kyoto, 606-8502 Japan

**Keywords:** dental reduction, enamel hypoplasia, Japanese, Jomon, Mesopotamian

## Abstract

Third molar (M3) agenesis has become prevalent since the terminal Pleistocene in humans. Population-level studies suggest that M3 agenesis is related to genetic factors and to environmental stresses such as malnutrition and infectious diseases. In addition, the fact that there are populations, such as the Jomon people, that have a high frequency of enamel hypoplasia (EH), an indicator of stress, but a low M3 agenesis rate, suggests that there are population differences in how the effects of environmental stress on M3 agenesis are manifested. In this study, I used EH to investigate the effects of environmental stress on M3 agenesis in three populations with different genetic bases: recent Japanese (1868–1926), Jomon people, and Islamic Mesopotamians. The results showed the effects of environmental stress on M3 agenesis in recent Japanese and Islamic Mesopotamians, but not in Jomon people. Furthermore, it was shown that M3 agenesis of recent Japanese and Islamic Mesopotamians are similarly susceptible to environmental stress. Thus, the results showed that the extent to which environmental stress affects M3 formation varies among populations. Since M3 agenesis was observed even in Jomon people, whose M3s are not thought to have been affected by environmental stress, it is reasonable to assume that M3 agenesis can be congenital or caused by environmental stress, and each cause of agenesis occurred independently in human populations.

## Introduction

In the early Holocene, the introduction of food production and new food preparation techniques (e.g. earth ovens, earthenware pottery, and eating utensils) reduced the necessity for vigorous mastication and caused teeth and jaws to reduce in size (Scott and Turner, 1988). The high prevalence of third molar (M3) agenesis observed in many modern human populations is not prevalent until the terminal Pleistocene (Lacy, 2021).

Besides modern humans, only Denisovans show a high frequency of M3 agenesis (Sawyer et al., 2015; Kaifu and Athreya, 2025; Tsutaya et al., 2025). The fact that most of the few recovered M2s and mandibles of Denisovans show M3 agenesis suggests that it was common among them (Sawyer et al., 2015; Kaifu and Athreya, 2025; Tsutaya et al., 2025). The M3 agenesis of Denisovans is a more peculiar case, given the strong masticatory forces expected from their robust mandibles and teeth (Tsutaya et al., 2025). However, as the number of Denisovan tooth and jawbone specimens found is small and their M3 agenesis is likely to be a unique case in human evolutional history, I will not delve into Denisovans in depth in this study.

The probable mutation effect (PME) is a well-known hypothesis to explain the degeneration or shrinkage of organs that are no longer functional (Brace, 1963, 1964). According to this hypothesis, structures that become functionally obsolete experience a relaxation in selective pressure, allowing neutral mutations to accumulate, which can lead to size reduction or eventual loss of the concerned structures (Brace, 1963). It has been shown that a reduction in human tooth size and number can occur through PME even in the absence of natural selection (McKee, 1984; Vukelic et al., 2017). However, there is a possibility that smaller teeth were naturally selected because teeth that are too large may have deleterious effects (Calcagno and Gibson, 1988).

From the genomic point of view, specific genomic regions have been found to be associated with M3 agenesis. A genome-wide association study conducted by Haga et al. (2013) identified three genomic regions showing evidence of association with M3 agenesis; the strongest association was found with the single-nucleotide polymorphism (SNP) rs1469622, which maps to the intron of THSD7B (Haga et al., 2013). SNPs rs938036 and rs906628 also showed evidence of association with M3 agenesis (Haga et al., 2013). In addition, it is considered that the formation of the M3 follicle is influenced by additive genetic factors (Trakinienė et al., 2018).

It is known that there are population differences in M3 agenesis rate (i.e. the proportion of individuals with M3 agenesis) among geographically and chronologically different human populations. Among modern humans, the lowest prevalence of M3 agenesis is in Australian Aboriginals and African populations, at around 1% and 6%, respectively. In comparison, it is around 20% in European and American populations, and the highest is around 25–30% in Middle Eastern and South and East Asian populations (King et al., 2010; Carter and Worthington, 2015). Among East Asian populations, the northern Sinodont populations have a higher M3 agenesis rate than the southern Sundadont populations (Turner, 1987). These populational differences suggest the involvement of genetic factors in M3 agenesis.

On the other hand, it is considered that environmental factors also play a crucial role in M3 agenesis. M3 is the last tooth to develop and erupt, and is considered to be more susceptible to environmental influence in its development than other teeth (Goodman and Armelagos, 1985; Yamada et al., 2004). Notably, M3s have a significantly greater morphological variation and a higher agenesis rate than other teeth (Lavelle and Moore, 1973). Even if M3s are formed in modern humans, it is not uncommon for them to not reach the occlusal surface (Carter and Worthington, 2016). This state is called impaction, and is considered to be caused by the underdevelopment of the maxilla and mandible and the resulting lack of space in the alveolar arch distal to the second molar (M2) (Osuga et al., 1999; Breik and Grubor, 2008; Kanazawa and Sakaue, 2018). Agenesis and impaction can often be confusing, but unlike M3 impaction, it is unlikely that M3 agenesis is influenced by the degree of jaw development. M3 tooth germ formation begins around 5 or 6 years of age (Tanaka et al., 1994; Masutomi et al., 2019), but jawbone growth continues until around the age of 20 (Love et al., 1990). Furthermore, in Japan, the incidence of malocclusion and crowding due to discrepancy has increased over time, which suggests a deterioration in the degree of jaw development (Hanihara et al., 1981; Inoue, 1990). However, despite this consistent trend, the M3 agenesis rate gradually increased until the early Showa era, and then decreased (Yamada et al., 2004). Details of the research on the changes in the M3 agenesis rate in the Japanese population are presented in the next paragraph.

Yamada et al. (2004) investigated the secular change in the frequency of M3 agenesis (i.e. the proportion of congenitally missing M3s to the total number of teeth surveyed) in the Japanese archipelago and reported that the frequency, which was less than 10% in the Jomon period, increased sharply to about 20% in the Yayoi period. This change is attributed to changes in gene frequency due to immigration from the continent (Yamada et al., 2004). This demographic change is explained by the dual-structure model of Hanihara (1991), which posits that modern Japanese people were formed by interbreeding between Jomon people and Yayoi immigrants. This model is supported by both morphological and genetic studies (Matsumura, 1994; Nakagome et al., 2015; Jinam et al., 2015; Kim et al., 2025). Recent studies suggest that large-scale population influx was completed by the time of the Kofun period (Cooke et al., 2021). Based on this, the change in the frequency of M3 agenesis among Japanese people since the Kofun period could be due to non-genetic factors. It is speculated that the increase in frequency of M3 agenesis from the Edo period to the early Showa era was due to malnutrition, and the decrease since the late Showa era was due to improvements in nutrition and health (Yamada et al., 2004).

Other reports also suggest the effects of environmental stress on M3 agenesis. In Chichester, UK, from the mid-16th century to the mid-19th century, the M3 agenesis rate (proportion of individuals with M3 agenesis) was as high as 42.7%, a prevalence unmatched in any clinical data from the UK (Caldwell, 2021). Historical records indicate that Chichester was plagued by epidemics of infectious diseases such as cholera and typhoid in the 19th century and even before that time, and it is speculated that such environmental stress may have contributed to M3 agenesis (Caldwell, 2021).

Tooth agenesis is generally understood to result from disruptions during the cell proliferation phase and is strongly influenced by genetic factors (Azzaldeen et al., 2017). The genetic basis of tooth agenesis is often explained by a polygenic model (Nakata, 1979; Matalova et al., 2008; Al-Ani et al., 2017). However, environmental influences should also be considered, as cell proliferation requires adequate nutrition (Kasuga et al., 2013; Pawar et al., 2021). Both Yamada et al. (2004) and Caldwell (2021) inferred environmental stress, such as malnutrition and infectious diseases at the population level, from the social background, but it is unclear whether individuals who had been subjected to environmental stress contributed to the high M3 agenesis rates. Observations of stress markers and morphological indicators that appear on bones and teeth are useful to infer the environmental stress at the individual level.

Carter (2016) used fluctuating asymmetry as a stress indicator to investigate whether environmental stress affects M3 agenesis. Fluctuating asymmetry (FA) is a developmental disorder that causes random deviations from bilateral symmetry in body parts (Van Valen, 1962; Naugler and Ludman, 1996). It is commonly used to estimate the effects of minor developmental accidents (Van Valen, 1962). Carter (2016) investigated the association between M3 agenesis and FA of the maxillary and mandibular morphology in pre-agricultural, transitional, and agricultural populations from Japan, Germany, Egypt, and America, and in pre-industrial, transitional, and post-industrial populations from Portugal and Japan, and found no association. The results obtained by Carter (2016) that did not support the hypothesis that M3 agenesis is caused by environmental stress may be influenced by the difference in the timing of the formation of the FA of the maxilla and mandible and M3. The FA of the jaw bones is determined prenatally (Gawlikowska-Sroka et al., 2017), whereas the formation of the M3 tooth germ begins at 5 or 6 years of age in the earliest individuals (Tanaka et al., 1994; Masutomi et al., 2019). That means FA of jawbone morphology does not indicate the stress that the individual experienced during the M3 tooth germ formation period. However, to my knowledge, no studies used indicators that indicate the stress an individual experiences during the M3 tooth germ formation period.

Enamel hypoplasia (EH) is one of the most commonly used stress markers, and indicates stress during the M3 tooth germ formation period (Fujita, 1995). EH results from impaired secretion of the enamel matrix after ameloblast differentiation and is primarily attributed to environmental factors such as malnutrition and infectious diseases (Wong, 2014). Certain populations, such as the Jomon people, have a high frequency of EH (Yamamoto, 1988), but a low frequency of M3 agenesis (Yamada et al., 2004). This may suggest there are populational differences in the effects of environmental stress on M3 agenesis, but no research has examined this.

Thus, to understand the effects of environmental stress on M3 agenesis, comparing groups with different stress levels within a population with the same genetic basis is appropriate. By performing this comparison among multiple populations with different genetic backgrounds and comparing the results between the populations, it is possible to clarify the population differences in how the effects of environmental stress manifest.

This study uses EH as an indicator of environmental stress to clarify the effects of environmental stress on M3 agenesis and its population differences. The reason for using EH is that it is formed from weaning to early childhood (Gawlikowska-Sroka et al., 2017) and is caused by nutritional deficiencies (in vitamin A, C, D, calcium, and phosphorus), metabolic disorders, infections, etc. (Martinhao et al., 2015). The crown formation periods of mandibular canines (LC) and maxillary central incisors (UCI), which are often used for EH observations, are from postnatal 4 or 5 months to 6 or 7 years and from 3 or 4 months to 4 or 5 years, respectively (Fujita, 1995). These are the same periods as or relatively near the start of M3 tooth germ formation (around 5 or 6 years: Tanaka et al., 1994; Masutomi et al., 2019), and therefore can provide information on the stress state during or near the M3 tooth germ formation period.

In this study, I examine two points: whether environmental stress as predicted from EH is associated with M3 agenesis, and whether there are populational differences regarding this association. Specifically, I predict that environmental stresses such as malnutrition and infection make individuals more susceptible to M3 agenesis, and that the more severe the environmental stress, the higher the proportion of individuals with M3 agenesis and the number of congenitally missing M3s. I also predict that the manifestation of the effects of environmental stress on M3 agenesis will differ depending on the population.

## Materials and Methods

The samples used in this study are composed of 218 recent Japanese (1868–1926), 53 Jomon, and 65 ancient Mesopotamian individuals (Table 1). Recent Japanese individuals include mainland Japanese from the Meiji to Taisho era (1868–1926) (The Kyoto University Museum collection). The Jomon individuals are those from the early to final Jomon period excavated from several sites across Japan (curated in the Laboratory of Physical Anthropology, Graduate School of Science, Kyoto University, and the Biological Anthropology Laboratory, Graduate School of Human Sciences, Osaka University). The ancient Mesopotamian individuals are skeletons from the Islamic period excavated from the Hamrin Basin in Iraq (housed in The Kyoto University Museum: Ishida et al., 2006). Only adults were surveyed. Individuals in which it was impossible to observe the presence or absence of M3 and EH were excluded from the samples. For individuals without age records, adulthood was determined based on the conditions of the spheno-occipital symphysis, clavicular epiphysis, palatine sutures, and cranial sutures, or the M3 eruption (at least one M3 was identified) (White and Folkens, 2000; Ikeda and Kinoshita, 2022).

**Figure 1.**
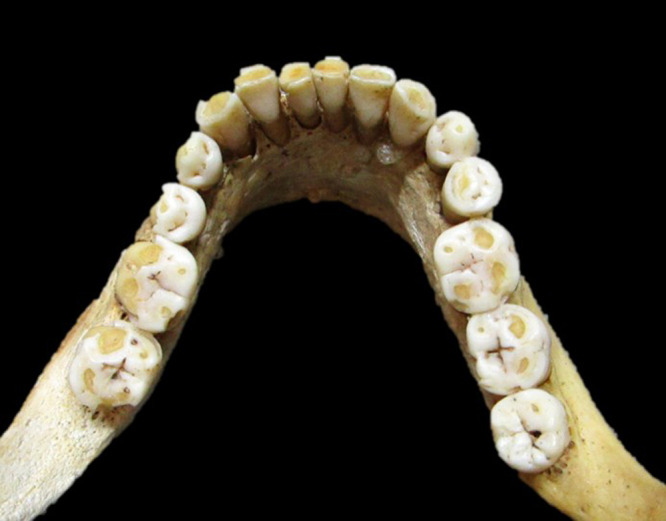
Islamic Mesopotamian individual with left mandibular M3 agenesis.

**Figure 2.**
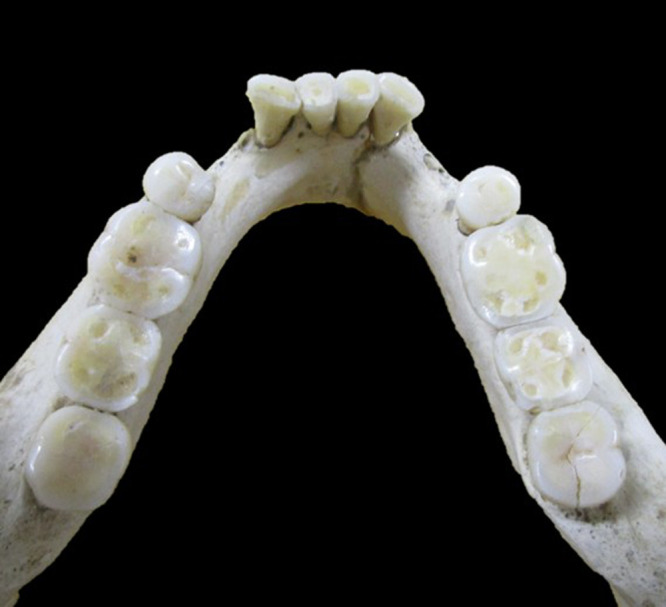
Jomon individual with bilateral mandibular M3s.

**Figure 3.**
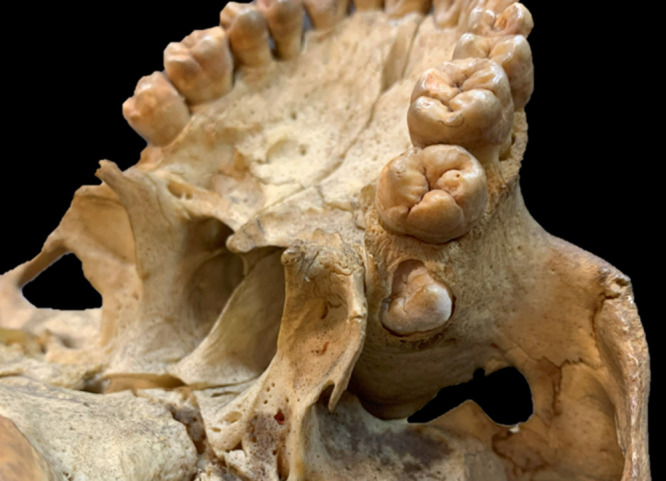
Forty-three-year-old recent Japanese individual: impacted M3 observed in the maxilla due to bone resorption.

The presence or absence of M3s was judged by visual observation (Figure 1, Figure 2). It is said that impaction does not significantly affect the results because the corresponding part of the alveolar bone is often resorbed, and M3s inside the bones can be observed even in individuals with M3 impaction (Kanazawa and Sakaue, 2018) (Figure 3). Therefore, X-rays were not used in this study. The criteria for M3 presence also include an alveolar socket at the location of the M3, a wear facet on the M2 distal crown surface, and attrition on the occlusal surface of the M3 (Yamada et al., 2004). If attrition is present on the occlusal surface of the M3, it suggests the presence of the opposing M3.

LCs are the most suitable for observing EH; UCIs can also be used (Goodman and Armelagos, 1985; Yamamoto, 1988). Therefore, LCs were used for EH observation in recent Japanese and Islamic Mesopotamians. However, LCs were often removed by ritual tooth ablation in Jomon people (Fujita, 2002). Therefore, if LCs were absent, UCIs were used for EH observation in Jomon specimens. To quantify the magnitude of environmental stress, EH symptoms were classified into three categories: linear (mild), pitting (intermediate), and grooved (severe) (Yamamoto, 1988; Witzel et al., 2006).

M3 agenesis rate and the average number of missing M3s per individual were calculated and compared between groups with and without EH and varying EH severity. Additionally, the frequency distribution of the number of missing M3s (1–4) in the M3 agenesis group was compared between those with and without EH. Damaged specimens may be used for individual counts for the absence/presence of M3 agenesis but not for calculating the number of missing teeth. A chi-square test was used to compare the rates of M3 agenesis, while Fisher’s exact test (two-sided) was applied when the sample size for the group was small. To evaluate the average number of missing M3s, a *t*-test (two-sided) followed the assessment of variance homogeneity using an *F*-test. Additionally, Fisher’s exact test (two-sided) was utilized to compare the frequency distribution of missing M3 numbers between the M3 agenesis groups with and without EH. A *P*-value of 0.05 or less was used as the significance level in all tests. To compare the degree of the effects of environmental stress on M3 agenesis between populations, Cohen’s *d* effect size was calculated in the *t*-test and used to analyse the difference in the average number of missing M3s between individuals with and without EH. The effect size serves as an index to assess the magnitude of the difference between the two groups, where an approximate effect size in the *t*-test of 0.20 indicates small, 0.50 indicates medium, and 0.80 indicates a large effect.

## Results

Table 2 presents the M3 agenesis rate, EH prevalence rate along with its breakdowns, and the comparison of M3 agenesis rates and the average number of missing M3s between the presence and severity of EH across different populations. The M3 agenesis rate was 50.9% in recent Japanese, 11.3% in Jomon, and 24.6% in Islamic Mesopotamians. In other words, the prevalence of M3 agenesis approximately doubled in the sequence: Jomon, followed by Islamic Mesopotamians, and then recent Japanese.

The EH prevalence rate was 63.3% in recent Japanese, 77.4% in Jomon, and 67.7% in Islamic Mesopotamians. Among those with EH symptoms, linear EH was 78.3%, and pitting EH was 21.7% in recent Japanese, linear EH was 100.0% in Jomon, and linear EH was 84.1% and pitting EH was 15.9% in Islamic Mesopotamians. No grooved EH was observed. In summary, there were no significant differences in the EH prevalence among the three populations, but the patterns of occurrence differed, with linear EH being most common in Jomon.

**Figure 4.**
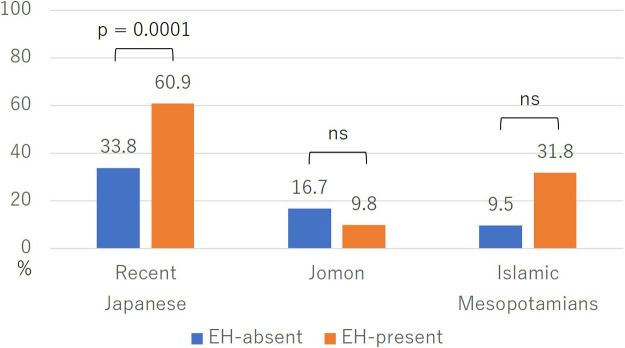
Comparison of M3 agenesis rates with and without EH. *P*-value of each population: recent Japanese, 0.0001; Jomon, 0.61; Islamic Mesopotamians, 0.07.

Figure 4 presents the comparison of M3 agenesis rates with and without EH. In recent Japanese and Islamic Mesopotamians, the M3 agenesis rate in the EH-present group tended to be higher than in the EH-absent group, but the difference was significant only in the recent Japanese comparison (*P* < 0.0001). In Jomon people, there was no tendency for the M3 agenesis rate to be higher in the EH-present group, and there was no significant difference (*P* = 0.61).

**Figure 5.**
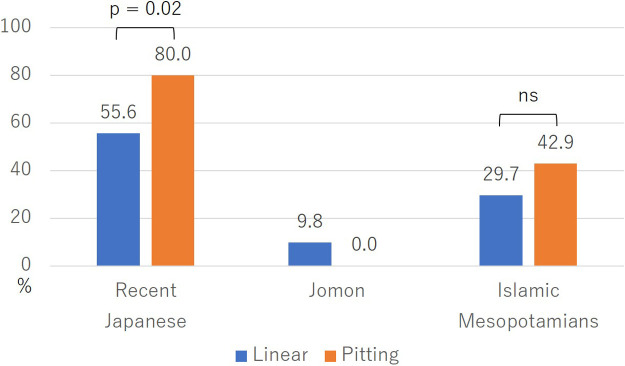
Comparison of M3 agenesis rates by EH severity. *P*-value of each population: recent Japanese, 0.02; Islamic Mesopotamians, 0.66.

Figure 5 presents the comparison of M3 agenesis rates by EH severity. In both recent Japanese and Islamic Mesopotamians, the M3 agenesis rate in the pitting group was higher than that in the linear group, but the difference was significant only in the recent Japanese comparison (*P* = 0.02).

A tendency for the average number of missing M3s to be higher in the EH-present group was observed in recent Japanese and Islamic Mesopotamians (Table 2). Both recent Japanese and Islamic Mesopotamians showed a significant difference (*P* < 0.0001, *P* = 0.02, respectively). In Jomon, the average numbers of missing M3s in the EH-absent and EH-present groups were 0.18 and 0.13, respectively, indicating no significant difference (*P* = 0.78).

When comparing the average number of missing M3s by EH severity, the average number of missing M3s in the pitting EH groups was higher in both recent Japanese and Islamic Mesopotamians, but only recent Japanese showed a significant difference (*P* = 0.02; Table 2).

**Figure 6.**
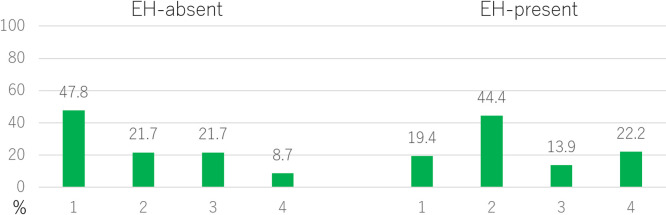
Frequency distribution of the number of missing M3s (1–4 teeth) in the Japanese M3 agenesis group. *P* = 0.02

Due to limitations in sample size, the only population for which the frequency distribution of missing M3s (1–4 teeth) in the M3 agenesis group could be compared between groups with and without EH was recent Japanese (Figure 6). In the EH-absent group, the most common number of missing M3s was one tooth, observed in 47.8% of individuals, followed by two and three teeth in 21.7% each, with four teeth being the least common, observed in 8.7% of individuals. In the EH-present group, the most common number of missing M3 was two (44.4%), followed by four (22.2%), and one and three (19.4% and 13.9%, respectively). The frequency distribution of the number of missing M3s among groups was significantly different (*P* = 0.02).

The Cohen’s *d* effect size for the difference in the average number of missing M3s resulting from the presence or absence of EH was 0.63 for recent Japanese and 0.67 for Islamic Mesopotamians, both categorized as medium effect sizes, while for Jomon, it was 0.10, indicating no effect (Table 3).

## Discussion

### Effects of environmental stress on M3 agenesis

In recent Japanese, the M3 agenesis rate in the EH-present group was significantly higher than in the EH-absent group, and the average number of missing M3s was also significantly greater in the EH-present group (Figure 4, Table 2). It is likely that the environmental stress experienced during the M3 tooth germ formation period influences M3 agenesis in recent Japanese. This finding aligns with Yamada et al.’s (2004) speculation that the increase in the frequency of M3 agenesis from the Edo period to the early Showa era was caused by malnutrition. The M3 agenesis rate in the pitting EH group, which is considered more severe, was significantly higher than in the linear EH group, and the number of missing M3s was also significantly greater (Figure 5, Table 2). It is likely that M3 agenesis is influenced by the severity of environmental stress. When comparing the frequency distribution of missing M3s in the M3 agenesis group between those with and without EH, the group with EH showed a higher number of missing M3s, which was statistically significant (Figure 6). This finding suggests that the number of missing M3s increases in recent Japanese individuals when exposed to environmental stress.

In Jomon people, there were no significant differences in the M3 agenesis rates or the average numbers of missing M3s between the groups with and without EH (Figure 4, Table 2). The M3 agenesis rate and the average number of missing M3s did not show a tendency to be higher in the EH-present group, indicating that even if Jomon people were subjected to environmental stress during the M3 tooth germ formation period, it did not result in M3 agenesis. As no pitting or grooved EH was observed, whether the results would have differed under higher stress levels remains uncertain.

In Islamic Mesopotamians, the EH-present group exhibited a higher M3 agenesis rate, although the difference was not statistically significant (Figure 4). Conversely, the average number of missing M3s was greater in the EH-present group, and this difference was statistically significant (Table 2). While there was a statistically significant difference in the number of missing M3s, the agenesis rates did not show significant differences, likely due to the small sample size. Nevertheless, the consistent tendency for M3 agenesis to occur more frequently in the EH-present group in both comparisons suggests that environmental stress during the M3 tooth germ formation period may have influenced M3 agenesis in Islamic Mesopotamians. The M3 agenesis rate in the pitting EH group (seven individuals)—indicative of more severe stress—was higher than in the linear EH group, and the count of missing M3s was also greater in the pitting EH group, yet the differences were not statistically significant (Figure 5, Table 2). These findings are similarly likely attributable to the small sample size. Without a larger population study, the impact of stress level on the rate and number of M3 agenesis remains unclear.

This study supported the hypothesis that environmental stress can lead to M3 agenesis in recent Japanese and Islamic Mesopotamians. Carter (2016) used the FA of jawbone morphology as an indicator of environmental stress and investigated the relationship between environmental stress and M3 agenesis, but could not demonstrate the relationship. The EH used in this study reflects the stress experienced relatively near or at the same time as the M3 tooth germ formation period and is considered more suitable for investigating the relationship between M3 agenesis and environmental stress. In addition, Carter (2016) also investigated early modern Japanese populations, so it is unlikely that the different results were due to population differences. This shows that when investigating the effects of environmental stress on morphogenesis, it is necessary to consider the formation period of the stress markers.

### Population differences and two different causes of M3 agenesis

The effect size of the difference in the average number of missing M3s between groups with and without EH, calculated to assess the extent to which environmental stress affects M3 agenesis, was medium for recent Japanese and Islamic Mesopotamians, but showed no effect for Jomon people (Table 3). These results indicate that there are population differences in how the impacts of environmental stress manifest in M3 agenesis. In Jomon people, who have a low M3 agenesis rate despite having a high prevalence of EH, there were no effect of environmental stress in M3 agenesis, as expected.

Agenesis of teeth other than M3 is very rare in humans (Lavelle and Moore, 1973), and there are no recorded cases of other primates and archaic humans exhibiting the same dental formula as humans that show a comparably high prevalence of M3 agenesis, except for Denisovans (Lavelle and Moore, 1973; Sawyer et al., 2015; Lacy, 2021; Kaifu and Athreya, 2025; Tsutaya et al., 2025). Although M3 agenesis is common in Denisovans, given that it was not common in ancient anatomically modern humans or Neanderthals (Lacy, 2021), it is likely that this trait is not ancestral but has emerged recently. Therefore, human M3 agenesis, which has been recorded since the terminal Pleistocene, is assumed to be a congenital trait caused by genetic factors, and emerged relatively recently in the modern human lineage. Additionally, some Holocene populations are considered to be susceptible to M3 agenesis under conditions of environmental stress.

The M3 agenesis rate in the Jomon sample used in this study was as low as 11.3% but showed no effect of environmental stress. This suggests that M3 agenesis in Jomon people is congenital, attributable to genetic factors. Similar to the Jomon people, there are other populations that display a high frequency of EH yet a significantly lower rate of M3 agenesis. For instance, the M3 agenesis rate in the North American hunter-gatherer population of Indian Knoll is 5.4% (Lacy, 2021), while the EH prevalence rate is as high as 89.2% (Perzigian, 1977). The M3 agenesis observed in these populations is considered to be congenital, and not caused by environmental stress. The M3 agenesis found in the EH-absent groups among recent Japanese and Islamic Mesopotamians is also considered congenital.

On the other hand, the inhibition of M3 formation caused by environmental stress is a more unusual phenomenon because it is not observed in all human populations like congenital M3 agenesis. The effects of environmental stress on M3 agenesis were observed in recent Japanese and Islamic Mesopotamians but not in Jomon people. Since congenital M3 agenesis was also observed in Jomon people, the inhibition of M3 formation by environmental stress likely occurred independently of congenital M3 agenesis. Japanese and Mesopotamians were agriculturalists (Bellwood, 2005; Miyamoto, 2019; Zeder, 2011; Vostretsov, 2021), whereas Jomon people were hunter-gatherers (Shitara, 2014). This leads to speculation that lifestyle differences influenced how environmental stress affects M3 agenesis. It is known that the transition from hunting and gathering to agriculture has led to various changes in human physical traits such as craniofacial gracilization and tooth size reduction (Larsen, 1995). The inhibition of M3 formation might have also appeared with the onset of agriculture. It may be part of the degeneration of the masticatory system resulting from the reduced need for vigorous mastication in agricultural populations (Larsen, 1995; von Cramon-Taubadel, 2011; Gkantidis et al., 2021), or a by-product of changes in other physical traits (Gould and Lewontin, 1979). The possible reason for Jomon people having low M3 agenesis rate despite having a high EH frequency rate is that the need for M3 was still high due to their hunter-gatherer lifestyle. Jomon people had well-developed jawbones, no impaction, few malocclusions, severe bite attrition, and edge-to-edge bite, which suggest that they had a diet that required vigorous mastication (Katayama, 2015). However, this hypothesis is founded on a study involving only three populations. Therefore, it must be validated by exploring the relationship between M3 agenesis and environmental stress in various regional and historical populations with diverse lifestyles and agricultural histories.

Population differences in both congenital M3 agenesis rates and the susceptibility of M3s to environmental stress may result from the differences in the degree of relaxation of selective pressure on M3s in each population due to their different lifestyles. Given that the rates of congenital M3 agenesis is not at or near 100% even in any agriculturalist population, and that M3 agenesis does not necessarily occur in response to environmental stress, the PME, rather than natural selection, would be a better explanation for human M3 agenesis. Even in populations where environmental stress can contribute to M3 agenesis, such as recent Japanese and Islamic Mesopotamians, M3 agenesis is not necessarily caused by environmental stress.

## Conclusion

The results obtained in this study indicate that environmental stress can lead to M3 agenesis in both recent Japanese and Islamic Mesopotamians. Additionally, the study demonstrates that there are population differences in the manifestation of the effects of environmental stress on M3 agenesis. Since M3 agenesis was observed even in Jomon people, whose M3s are unlikely to be affected by environmental stress, it is reasonable to assume that M3 agenesis is congenital or is caused by environmental stress as in recent Japanese and Islamic Mesopotamians.

## Figures and Tables

**Table 1.** Materials

Population	Area	Period	N
Recent Japanese	Mainland Japan	1868–1926	218
Jomon	Kawachikou, Osaka	Early Jomon	3
	Hosoura, Iwate	Middle Jomon	1
	Tsukumo, Okayama	Late and terminal Jomon	31
	Yoshigo, Aichi	Terminal Jomon	15
	Inariyama, Aichi	Terminal Jomon	3
	Total		53
Islamic Mesopotamians	Hamrin basin, Iraq	Islamic period	65
Total			336

All individuals are adults.

**Table 2.** Results

	Recent Japanese	Jomon	Islamic Mesopotamians
M3 agenesis rate	50.9% (111/218)	11.3% (6/53)	24.6% (16/65)
EH prevalence rate	63.3% (138/218)	77.4% (41/53)	67.7% (44/65)
Linear EH	78.3% (108/138)	100% (41/41)	84.1% (37/44)
Pitting EH	21.7% (30/138)	0.0% (0/41)^a^	15.9% (7/44)
Grooved EH	0.0% (0/138)	0.0% (0/41)	0.0% (0/44)
EH-absent group M3 agenesis rate	33.8% (27/80)***	16.7% (2/12)	9.5% (2/21)
EH-present group M3 agenesis rate	60.9% (84/138)	9.8% (4/41)	31.8% (14/44)
Linear group M3 agenesis rate	55.6% (60/108)*	9.8% (4/41)	29.7% (11/37)
Pitting group M3 agenesis rate	80.0% (24/30)	—	42.9% (3/7)
EH-absent group average missing M3s	0.58***	0.18	0.14*
EH-present group average missing M3s	1.34	0.13	0.71
Linear group average missing M3s	1.19*	0.13	0.69
Pitting group average missing M3s	1.92	—	0.83

^a^ No pitting EH was observed in the Jomon sample. *P < 0.05, **P < 0.01, ***P < 0.001.

**Table 3.** Cohen’s d effect size in each population

Population	Effect size
Recent Japanese	0.63
Jomon	0.10
Islamic Mesopotamians	0.67

Classification of effect size: 0.20, small; 0.50, medium; 0.80, large. Recent Japanese and Islamic Mesopotamians showed a medium effect size.
